# Decreasing trend of monkeypox cases in Europe and America shows hope for the world: Evidence from the latest epidemiological data

**DOI:** 10.1002/hsr2.1030

**Published:** 2022-12-29

**Authors:** Md. Robin Khan, Md. Jamal Hossain, Arpita Roy, Md. Rabiul Islam

**Affiliations:** ^1^ Department of Pharmacy University of Asia Pacific Dhaka Bangladesh; ^2^ Department of Pharmacy State University of Bangladesh Dhaka Bangladesh; ^3^ Department of Biotechnology, School of Engineering & Technology Sharda University Greater Noida India

**Keywords:** disease outbreaks, epidemiology, monkeypox, monkeypox virus, mpox

## 1 BACKGROUND

Monkeypox (MPOX) is a zoonotic viral disease caused by an Orthopox DNA virus named mpox virus (MPOXV). Scientists first discovered MPOXV in a monkey transported from Singapore to Denmark for research purposes. However, at first, the virus's primary animal reservoir was rodents. In 1970, a 9‐year‐old child from the Democratic Republic of the Congo was first diagnosed with MPOX. Since then, 11 African nations have recorded human instances of MPOX.^1^ Between 1970 and 1979, six African countries reported only 48 confirmed human MPOX cases. However, African nations reported more than 400 human MPOX cases at a death rate close to 10% by 1986. Several minor MPOX outbreaks between 1991 and 1999 reported 500 MPOX cases in tropical Central and Western Africa.^2^ However, isolated cases have been discovered in countries outside of Africa since 2003. The United States of America (USA) had the first outbreak of MPOX outside of Africa in 2003.^3^ The multicountry MPOX outbreaks have been reported in nonendemic nations starting in early May 2022.^4^ Belgium, Sweden, and Italy detected their first confirmed MPOX cases on May 19, 2022. Australia reported the first case on May 20, 2022, in Sydney and Melbourne in persons who had visited Europe just before their detection. On May 20, 2022, France, Germany, and the Netherlands reported their first cases. In contrast, the United Arab Emirates announced the finding of its first case in late May 2022.^2^ Spain reported its first two deaths in July 2022, and Belgium reported its first death in August 2022 due to MPOX disease.^5^ On July 23, 2022, the World Health Organization (WHO) declared MPOX a Global Public Health Emergency.^6^ Even though the MPOX disease has a low fatality rate ranging from 3% to 6%, the recent outbreak, and its epidemiological data have created concern among the general population worldwide. MPOX signs and lesions can be hard to tell apart from smallpox in their clinical presentation.^7^ Backache, headache, chills, fever, weariness, myalgia, lethargy, and swollen lymph nodes are some of the nonspecific symptoms of MPOX disease that first appear. Three days later, the fever goes down and the rash begins to centrifugally cover the body. Similar to the smallpox rash, it begins as macules for 2–4 weeks before changing into papules, vesicles, pustules, and lastly crusts and scabs.^7^ With sizes ranging from 0.5 to 1 cm, the numbers can approach the thousands.^7^ They originate from the trunk and disperse throughout the body in a centrifugal pattern. MPOX can be distinguished from other infections diseases by the severe lymph node enlargement that is seen in the neck, axillary, and groin areas.^7^ There may also be pharyngeal, conjunctival, and vaginal mucosal inflammation. Numerous issues have been documented, including secondary bacterial infections, respiratory issues, bronchopneumonia, encephalitis, corneal infections with subsequent vision loss, gastrointestinal issues, vomiting, and diarrhea with dehydration.^8^ MPOX continues to primarily affect young men who have sex with men (MSM), between 18 and 50 years (87%). The overall risk of MPOX infection is estimated as moderate for MSM and low for the general population based on data from the present outbreak.^9^ However, the smallpox vaccine showed protection in 85 cases out of 100 cases of MPOX.^10^ MPOX‐related hospitalization remains rare; studies indicate that the proportions range from 5% to 10%.^11^ Hospitalization may be necessary for patients who experience complications from MPOX, with the most frequent causes being severe anorectal and genital pain that needs analgesia, bacterial superinfection (cellulitis) affecting the genital and perineal region, urinary retention due to penile edema, or ocular involvement. A severe or complicated MPOX infection, such as the emergence of a more extensive rash, may affect patients who are pregnant, children, or those who are immunocompromised, such as those with advanced or untreated HIV.^11^

## 2 THE PRESENT SITUATION OF THE MPOX OUTBREAK

The majority of cases reported in the recent multicountry outbreak were reported from the Region of the Americas (66.9%) and the European Region (31.4%).^12^ Most MPOX cases recorded in Europe and America were individuals who were identified through primary care and sexual health services, but who had no prior history of travel to the African countries where the disease is widespread. Although there has never been any evidence that MPOX is sexually transmitted, the majority of instances recently reported have included homosexual, bisexual, or other males who have intercourse with men. The MPOX virus was reportedly also found in the infected patients' semen.^13^ So it's possible that MPOX could spread through close physical contact during sexual activity. Due to societal and religious issues, the LGBTQI+ community does not have equal rights in many Asian nations compared to Europe and America.^13^ May be this is playing a vital role in the recent outbreak of MPOX virus in America and Europe. Between January 1 and October 30, 2022, WHO reported 77,264 laboratory‐confirmed MPOX cases and 36 fatalities from 109 countries or territories. Among the MPOX cases, 86.4% have been detected by the 10 European and American nations.^11^ The USA reported the highest MPOX cases (28,379), followed by Brazil (*n* = 9162), Spain (*n* = 7317), France (*n* = 4094), the United Kingdom (*n* = 3698), Germany (*n* = 3662), Colombia (*n* = 3298), Peru (*n* = 3048), Mexico (*n* = 2654), and Canada (*n* = 1437). However, MPOX cases have been on a general decline since August 2022. The number of new cases globally has decreased by 40.7% in the 43rd week (between 24 and October 30, 2022) compared to the previous week (1295 vs. 2182).^11^ The countries in America (−41%) and Europe (−38%) together reported the maximum decrease in new MPOX cases.^11^ In a similar vein, the number of new cases reported each week across the globe decreased by 17% in week 45 (7 through 13 November) (*n* = 1114 cases) compared to week 44 (31 October through 06 November) (*n* = 1348 cases), with the Region of the Americas experiencing the largest proportional decline (−20%).^12^ As of November 22, 2022, 15 European nations had gone more than 21 days without reporting an MPOX case. The number of newly reported cases during week 43 has decreased by 98.1% when compared to the peak of reported cases (2163 cases during week 29; 18–24 July 2022).^14^ Recent WHO data demonstrate a global trend toward a decrease in the number of new cases of MPOX reported; this decrease was 3.7% between weeks 45 (07 November–13 November) and 46 (14 November–20 November), with 1090 cases reported in week 46 and 1132 cases reported in week 45.^14^

## 3 POSSIBLE REASONS FOR DECREASING TREND OF MPOX CASES

There could be several reasons why MPOX instances have recently decreased worldwide, particularly in Europe and America. Increasing immunity in the most affected population groups due to natural immunity and vaccination, behavioral changes, and a significant decrease in the number of cultural and social activities after the summer, especially by the key risk groups (MSM) for this outbreak, are some of the most crucial factors, along with risk communication and community engagement.^9^ According to a US Centers for Disease Control and Prevention (CDC) study that surveyed men who have sex with men, almost half have been reducing risky sexual activity due to virus fears.^15^ As MPOX can spread from person to person, it can be prevented by taking public health precautions such as early case detection, diagnosis and care, isolation and contact tracing, and self‐monitoring by contacts. Many international and national healthcare organizations such as the WHO, Center for Disease Control (CDC), and European Centre for Disease Prevention and Control (ECDC) regularly share updated learning materials and guidelines on MPOX disease. International and national healthcare authorities have created awareness about MPOX risk factors, transmission, symptoms, remedies, and preventive measures.^16^ Therefore, people across the countries are now more aware of MPOX disease and practicing safety measures than at any previous time. The above initiatives have produced this positive indication in the new cases during the ongoing MPOX outbreak. In addition, the WHO member states have activated a public health response system for effective coordination of case finding, contact tracing, laboratory investigation, supported isolation, clinical management, infection prevention measures, risk communication, community engagement, and vaccination activities.^11^ Also, the WHO is continuously monitoring and publishing up‐to‐date epidemiological data on ongoing multi‐country MPOX outbreaks to help the member states and partners. The health authorities in the UK have established an incident management team to monitor the cases or contacts. Higher‐risk contacts like gay, bisexuals, men who have sex with men (MSM), multiple casual sexual partners, sex workers, lab staff working with orthopoxviruses, and clinical laboratory and healthcare workers doing diagnostic tests for MPOX, can choose to receive the vaccination.^17^ Apart from this, they advise self‐isolation for MPOX cases. The 3‐week quarantine for MPOX patients was introduced in many European and American countries.^2^

## 4 FURTHER MEASURES TO HASTEN THE DECREASING TREND OF MPOX CASES

According to the present epidemiological information, men who have sex with menare a high‐risk group for MPOXV spreading. Therefore, public health advice for men who have sex with menshould avoid these behaviors and public gatherings in this circumstance. However, so far in Europe and America, the majority of reported patients have been guys who have sex with men, though this is not true of all of them. It's crucial to remember that no one demographic is predisposed to contracting the MPOX virus. Stigmatizing particular communities hinders the public health response, which can further exacerbate the crisis, as we have repeatedly seen in situations as diverse as HIV/AIDS, TB, and COVID‐19. Early diagnosis, isolation, efficient contact tracing, and vaccine initiatives may also be crucial to hasten the decreasing trend of MPOX cases.^18^ According to study findings, vaccination and antiviral agents against smallpox may protect against MPOXV and lessen the severity of symptoms.^19^ Smallpox vaccination is advised by the Centers for Disease Control (CDC) for high‐risk contacts within 4 days and up to 14 days of contact. If administered between 4 and 14 days after the date of exposure, vaccination may lessen disease symptoms but will not prevent the illness.^20^ Due to the limited availability of vaccines, primary preventive vaccination (PPV) and postexposure preventive vaccination strategies may be used to specifically target those who are at a greater risk of exposure and close contact with cases.^9^ PPV initiatives should pay special attention to gay, bisexual, or other males or transgender people who have sex with men who are more likely to be exposed, as well as people who are at risk of exposure at work, based on epidemiological or behavioral criteria.^9^ Health promotion programs and community involvement are also essential to ensuring effective outreach and high vaccine acceptance and uptake among those most at risk of exposure.^9^ Immune globulin is another option for prophylaxis for patients with severe immunosuppression in addition to the smallpox vaccine, albeit its advantages are still unknown.^20^ It is possible to employ antiviral medications authorized to treat smallpox, such as tecovirimat and brincidofovir, to cure MPOX. Recently, for the treatment of MPOX, Tecovirimat has received approval from both the UK Medicines and Health Care Products Regulatory Agency and the European Medicines Agency.^12^ Therefore, mass vaccination by smallpox jabs and simultaneous use of supportive antiviral therapy for a high‐risk population may be an alternative to curb the ongoing MPOX outbreak. The clinical manifestations of the current outbreak, which are crucial for the typical practitioner to diagnose the disease, are another essential element that needs to address with importance. Healthcare professionals from all specialties should continue to gather information regarding the variety of case presentations, testing recommendations and testing methods, guidance on infection prevention and control in primary care, information on national public health initiatives, and risk communication strategies. Also, nations should assess their diagnostic capabilities and expand access to testing the trend of this outbreak. Moreover, contact and droplet infection control precautions for individuals with suspected or confirmed MPOX cases can be effective measures to curb the infection rate. The samples from individuals suspected of having MPOX or from animals infected with the MPOXV requires trained personnel operating in well‐furnished laboratories.

Any illness that develops while traveling or after leaving an area where it is endemic should report to a medical provider, along with details about any recent travel and immunization records to prevent further spread. Residents and visitors to endemic countries should avoid contact with sick or dead rodents, marsupials, or primates that may be carrying the MPOXV and should abstain from eating or handling wild meat. It is also crucial to stress the value of washing hands with soap and water or an alcohol‐based hand sanitizer among general people. The COVID‐19 pandemic has devastated lives and healthcare authorities worldwide.^21^, ^22^, ^23^, ^24^, ^25^ The recent multicountry MPOX outbreak has worsened the situation.^26^, ^27^, ^28^, ^29^ Therefore, the twin effect of the COVID‐19 pandemic and the MPOX outbreak is a global challenge for public health.

## 5 CONCLUSION

The recent downward trend of MPOX cases shows hope in the ongoing multicountry MPOX outbreak. The authorities should identify the associated factors behind this decreasing epidemiological trend of the MPOX outbreak to accelerate the rate. The traditional measures applicable for Covid‐19 might not be effective in this case because people now feel bored with following health safety guidelines for the past two and half years. Therefore, increasing the knowledge and awareness about the disease and providing the right message about the outbreak might bring positive change in their attitude and practice toward MPOX. Also, the healthcare authorities should check the rationality of mass vaccination of existing smallpox vaccines among the high‐risk population, and the researchers should search for target‐specific vaccines for MPOX disease.

## AUTHOR CONTRIBUTIONS

**Md. Robin Khan**: conceptualization; writing—original draft. **Md. Jamal Hossain**: writing—original draft. **Arpita Roy**: writing—review & editing. **Md. Rabiul Islam**: conceptualization; writing—review & editing.

## CONFLICTS OF INTEREST

The authors declare no conflicts of interest.

## TRANSPARENCY STATEMENT

The lead author Md. Rabiul Islam affirms that this manuscript is an honest, accurate, and transparent account of the study being reported; that no important aspects of the study have been omitted; and that any discrepancies from the study as planned (and, if relevant, registered) have been explained.

## Data Availability

Data sharing is not applicable to this article as no new data were created or analyzed in this study.
